# Cementless hemiarthroplasty for femoral neck fractures in elderly patients

**DOI:** 10.4103/0019-5413.38582

**Published:** 2008

**Authors:** Yusuf Öztürkmen, Mahmut Karamehmetoğlu, Mustafa Caniklioğlu, Yener İnce, İbrahim Azboy

**Affiliations:** İstanbul Training and Research Hospital, 2^nd^ Department of Orthopaedics and Traumatology, Turkey

**Keywords:** Cementless femoral prosthesis, cementless hemiarthroplasty, femoral neck fracture, hemiarthroplasty in elderly

## Abstract

**Objectives:**

The use of cement is associated with increased morbidity and mortality rate in elderly patients, hence cementless hemiarthroplasty is suggested. We evaluated the results of cementless hemiarthroplasty for femoral neck fractures in elderly patients with high-risk clinical problems.

**Materials and Methods:**

Forty-eight patients (29 females, 19 males) with a mean age of 88 years (range: 78 to 102 years). having femoral neck fractures were treated with the use of cementless hemiarthroplasty. Porous-coated femoral stems were used in 30 patients (62%) and modular type femoral revision stems in 18 patients (38%). Bipolar femoral heads were used in all patients. Radiological follow-up after operation was done at the one, three, six months and annually.

**Results:**

The mean follow-up period was 4.2 years (range: 18 months to eight years). None of the patients died during hospitalization. Medical complications occurred in six patients (12%) within the follow-up period and four patients (8%) died within this period. Only two hips were converted to total hip arthroplasty due to acetabular erosion. Femoral revision was planned for one patient with a subsidence of > 3 mm. None of the patients had acetabular protrusion or heterotopic ossification. The mean Harris-hip score was 84 (range: 52 to 92). Dislocation occured in one patient (2%).

**Conclusion:**

Cementless hemiarthroplasty is a suitable method of treatment for femoral neck fractures in elderly patients with high-risk clinical problems especially of a cardiopulmonary nature. This method decreases the risk of hypotension and fat embolism associated with cemented hemiarthroplasty.

## INTRODUCTION

The number of femoral neck fractures increases as age advances. Surgery is the mainstay of treatment for displaced femoral neck fractures, hemiarthroplasty being a common operation in elderly patients. Hemiarthroplasty is an easy surgical procedure, with shorter surgery time and less blood loss. Cemented prostheses are used more frequently but the possible effects of cement on the cardiopulmonary system and the greater technical challenge to revision of cemented prosthesis has led surgeons to prefer uncemented implants. Whether or not to cement the hemiarthroplasty is a perennial argument.^1^–^23^ In an attempt to resolve this we report the results of cementless hemiarthroplasty in elderly patients with femoral neck fractures.

## MATERIALS AND METHODS

**Figure 1(a-b):**
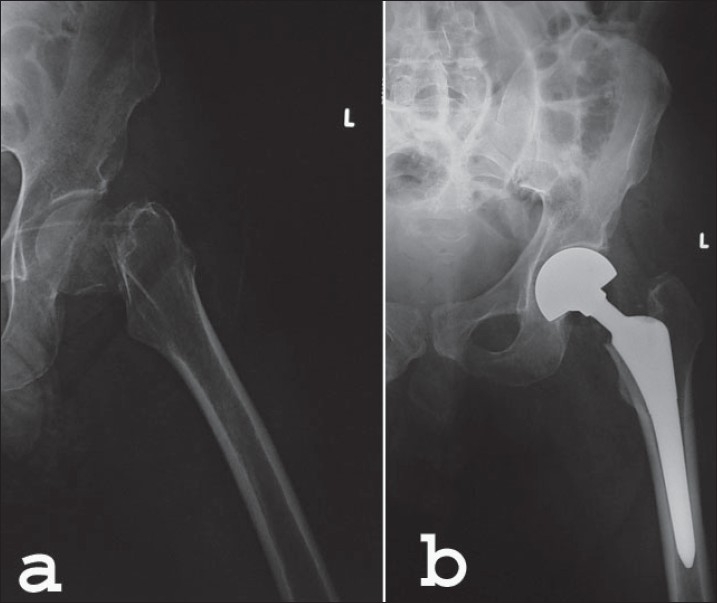
(a) Preoperative radiograph of a 88 year-old woman with a femoral neck fracture and (b) immediate postoperative X-ray of the same patient where cementless hemiarthroplasty is performed with the use of a porous-coated femoral stem and bipolar head. The fitting and filling of the medullary cavity is well visualized

**Figure 2 (a-b):**
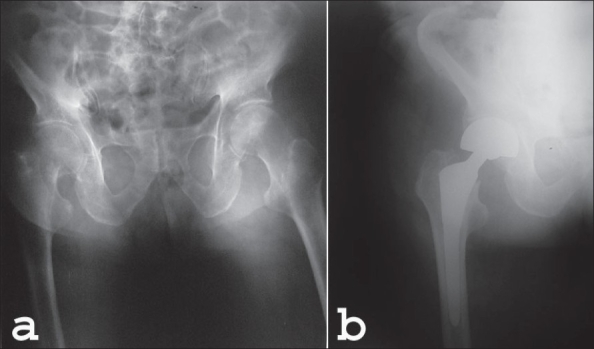
(a) Preoperative pelvis radiograph of a 84 year-old female with a right femoral neck fracture. (b) Followup X-ray of the same patient at 3 months. The initial filling and fitting of the medullary canal of the femur is good enough on the early postoperative radiograph

Between January 2001 and May 2006, we performed 48 cementless hemiarthroplasty operations on elderly patients. All patients (29 female and 19 male) were of, mean age 88 years (range: 78-102 years). All patients had displaced intracapsular femoral neck fractures (Garden^18^ stages III, IV). Out of 48 patients, 30 had porous-coated femoral stems (F 40 ergosystem, Biomet, Europe) and 18 had modular-type revision stems (Helios, Biomet, Europe) [Figures 1,2]. Bipolar femoral heads (Biomet, Europe) of sizes between 42 and 60 mm were used in all patients. All of the patients were able to ambulate before the initial trauma but ten (20%) of them needed support to walk. All patients were grade III or grade IV according to the American Society of Anaesthesiologists (ASA)^19^ classification. All the patients had serious medical comorbidities with high risk of mortality [Table 1]. Patients with a history of previous hip injuries and preexisting osteoarthritis were not included in the study group. The operation is done with the posterolateral approach after placing the patient in the lateral position. After dissecting the subcutaneous tissues, the fascia is divided in line with the skin incision over the center of the greater trochanter. The gluteus maximus fibers are splitted bluntly. Trochanteric bursa is excised. The sciatic nevre is palpated. The short external rotators are dissected while protecting the nerve. The capsule is divided with a T-incision but the entire capsule is preserved for later repair. After removing the femoral head, the hip is gently flexed, adducted and internally rotated. The femoral canal is reamed with increasing sizes of the reamers. After cortical reaming is felt, broaches are placed precisely. The fit of the broach within the canal is assessed. Adequate axial and rotational stability is tested with no motion of the broach in the canal. The selected porous-coated femoral stem is inserted. The precise size of the femoral stem is also tested to rotational and extraction forces. After inserting the predetermined and measured femoral bipolar head, the hip is reduced and the stability of the hip joint is again tested. The capsule is repaired firmly. After control of the bleeding vessels, the dissected soft tissues are repaired to finish the operation. The patients received prophylactic antibiotics for three days. Depending on the general status of the patient, supervised therapy for sitting, standing and walking with toe touch weight bearing was begun on the first postoperative day. The patient remained hospitalized until he or she was ambulating with crutches or walker. Elastic stockings were used throughout the hospitalization period. The patients were given instructions and occupational aids to avoid flexing the hip beyond 90°. Skin sutures were removed on the fifteenth postoperative day.

**Table 1:** Preoperative medical co-morbidities of our patients

	Number of patients
Cardiovascular disease	34
Diabetes mellitus	25
Chronic renal insufficiency	25
Chronic lung disease	27
Neuromuscular disease	5

34 patients had at least two of these complications

Full weight-bearing was allowed usually after six weeks when patient was comfortable and painfree. Patients were both clinically and radiographically evaluated during the postoperative follow-up period. Clinical evaluation was performed using the Harris^20^ hip scoring system. Pain was evaluated using a visual analogue scale. A score of 0 represented virtually no pain while a score of 1 to 5 represented mild, nonpersistent pain. A score of 5 to 8 represented moderate, persistent pain and a score > 8 corresponded to debilitating pain. Mobility was assessed according to the criteria of Bezwada *et al,*^5^ which were: ambulation without assistance, ambulation with the assistance of either a cane or a walker and the ability to climb and descend stairs. Radiographic evaluation included anteroposterior pelvis and lateral hip roentgenograms obtained at regular postoperative intervals: six weeks, three months, six months, one year and then annually. Femoral stem stability and fixation were assessed by the method of Engh *et al.*^3^ Femoral stem subsidence was defined as any change of position > 3 mm as demonstrated on serial radiographs. Femoral stems were classified as stable with bone growth, stable with fibrous ingrowth or unstable. Mechanical stem failure was defined as femoral stem revision for loosening or a radiographically unstable femoral component. Acetabular protrusion and erosions were radiographically evaluated by the method described by LaBelle *et al*.^3^ Acetabular protrusion was determined by measuring the medialization of the acetabular line as compared to the immediate postoperative radiograph with reference to Kohler's line. Acetabular cartilage erosion was assessed by measuring the change in thickness of the acetabular cartilage compared with the immediate postoperative radiograph or comparing to the normal hip joint cartilage. Joint space narrowing was evaluated as an acetabular cartilage erosion and this was measured in millimeters. Heterotopic ossification was graded according to Brooker's^21^ classification. All patients received low-molecular weight heparin postoperatively. The administered dose was 4000 anti-Xa IU and was initiated 12 hours before the operation and continued for ten days postoperatively.

## RESULTS

The mean operating time was 48 ± 20 minutes while the mean follow-up period was 4.2 years (range: 18 months to six years). None of the patients died during the postoperative hospitalization period. Perioperative medical complications are shown in Table 2. Four patients (8%) died within the follow-up period due to myocardial infarctions and renal insufficiency. Only two hips (4%) were converted to total hip arthroplasty due to acetabular erosion. One patient had a one mm joint space narrowing and the other one had a 2 mm of narrowing. Femoral revision was planned for one patient with a subsidence of > 3 mm. The other femoral stems were radiographically stable and demonstrated signs of bone ingrowth in accordance with the classification system of Engh *et al*.^16^ None of the patients had acetabular protrusion or heterotopic ossification.

**Table 2:** Perioperative medical complications

	Number of patients
Congestive heart failure	2
Deep venous thrombosis	2
Myocardial infarction	2
Pulmonary embolism	0

At the final follow-up, the mean Harris-hip score was 84 (range: 52 to 92). The pain score was 0 in 34 patients (71%), between 1 and 4 in ten patients (21%), between 5 and 8 in two patients (4%) and pain score >8 in two patients (4%). Fourteen patients (30%) were mobilized with crutches or walkers. Five patients (10%) were unable to ascend or descend stairs independently without the use of aids. These patients were not bedridden or confined to a wheelchair before the operation but they had a sedentary and minimum capacity for walking or other activity. The other patients were ambulatory without support although 19 (65%) of them needed an assistance device for a long walk. Complications observed were one dislocation and one superficial infection which responded to parental antibiotherapy. Closed reduction under general anesthesia was performed for the dislocated hip.

## DISCUSSION

Cemented femoral fixation of hemiarthroplasty has been the accepted technique for the treatment of displaced femoral neck fractures in elderly patients with a low life expectancy and poor bone stock. The cemented fixation provides immediate stability and allows immediate weight-bearing. This also improves the postoperative general status of the patient.^1^–^11^ However, cemented femoral technique has also been associated with a greater risk of fat embolization and hypotension. Christie *et al.*,^11^ have shown that femoral hemiarthroplasty with cement fixation is associated with more frequent and more extensive thromboembolic cascades than if cement is not used. Clark *et al.*,^12^ have also demonstrated significant falls in cardiac output and stroke volume during cementation.

More surgeons are now using porous-coated cementless fixation because of improved clinical results with modern implants and substantially reduced perioperative morbidity.^2^,^9^,^17^,^24^–^28^ Significant differences in the mortality rates have been reported for cemented and cementless hemiarthroplasty. Holt *et al.,*^17^ reported a mortality rate of 14.8% in patients with a cemented hemiarthroplasty compared to 9.8% in the cementless group. The difference was statistically significant. The use of acrylic cement was associated with increased morbidity and mortality rates in hemiarthroplasties. Neither the grade nor the experience of the operating surgeon had any effect on mortality and morbidity. Muirhead-Allwood^22^ also reported mortality rates of 15% in cemented hemiarthroplasty and 7% in cementless hemiarthroplasty during the first five weeks of follow-up and the reoperation rates were 13% for the cemented group and 6% in the uncemented group. We also observed similar results in our hospital. We used to prefer cemented hemiarthroplasty but patients were observed to die peroperatively or postoperatively during hospitalization due mostly to cardiovascular problems. However, in our cementless patient group there were no intraoperative or postoperative deaths during the hospitalization period. Only four patients (8%) died within the follow-up period. We believe that the improvement in mortality rate is probably due to a combination of improved anesthesia methods, medical treatment and the cementless technique itself.

One of the drawbacks of cementless hemiarthroplasty is component instability or the risk due to the lack of osteointegration affected by the poor bone quality of the elderly patient. However, while cemented fixation in older patients has not been shown to provide improved fixation, durability or long-term function over current cementless designs, it has also been associated with a possible increase in intraoperative pulmonary problems and cardiac arrest.^11^,^12^ Poor bone quality was not regarded as a contraindication and stable fixation of the porous-coated components is achievable in osteoporotic bones, which has also been reported by McAuley *et al.*^29^ and Cracchiole *et al.*^30^ In our study, we found that the initial filling and fitting of the medullary canal of the femur are very important at the time of femoral stem insertion for successful outcomes. This can be achieved with optimal preparation of the femoral medullary cavity.^23^,^24^,^27^ In cases when it was very difficult to fill the medullary canal, we preferred modular-type revision femoral stems. There are also some reports about the use of modular femoral prostheses in the treatment of pertrochanteric fractures and also for primary total hip arthroplasty in patients older than 70 years. Proximal and distal femoral canals can be adjusted so that an anatomic fill can be achieved even in patients with enlarged medulla.^9^,^15^,^28^ No embolism in these patients have been reported. We also used modular stems for patients in whom the porous-coated femoral stems failed to fill the medullary canal. We believe that the initial filling is very important to prevent later stem migration and that is why we had only one femoral stem subsidence. Elderly patients with a high activity level and good general health show a high migration of the stem with a solid bipolar femoral head.^6^ Total hip arthroplasty should be preferred for these patients. Hemiarthroplasty is advocated for elderly patients with a low life expectancy.^23^–^27^

It has been reported that patients treated with cementless hemiarthroplasty experienced greater hip pain than patients undergoing cemented hemiarthroplasty.^1^–^3^,^5^–^7^ However, there are also studies reporting no significant difference in pain scoring between the two groups postoperatively.^5^ Pain was not a major problem in our study group although the increased use of walking aids after cementless fixation is another drawback of this method. In our study, 10% of the patients were unable to ascend or descend stairs independently without the use of aids. However, all these patients also had some other medical comorbidities such as neurological disorders. The average operating time for cementless hemiarthroplasty was reported to be shorter than for cemented hemiarthroplasty.^7^ However the difference was not statistically significant. We also observed shorter operating times in our patients. We believe that shorter operating time with cementless hemiarthroplasty decreases the risks of anesthesia.

In conclusion, our study shows favorable results for elderly patients undergoing cementless hemiarthroplasty for displaced femoral neck fractures. The weak point of this study is the short follow-up time, hence, we intend to continue follow-up to evaluate long-term results. In view of the shorter operating times and low mortality rates due to the absence of cement-related complications, cementless hemiarthroplasty may be of particular benefit in elderly patients with high-risk cardiopulmonary problems.
